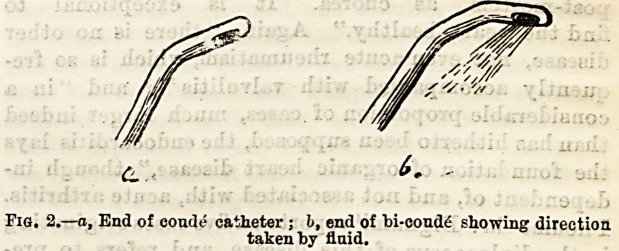# The Treatment of Chronic Cystitis

**Published:** 1895-03-30

**Authors:** G. Munro Smith

**Affiliations:** Senior Assistant Surgeon, Bristol Royal Infirmary


### THE TREATMENT OF CHRONIC CYSTITIS.

By G. Munro Smith, Senior Assistant Surgeon, Bristol Royal Infirmary.

In the common forms of chronic cystitis—associated with enlarged prostate, stricture, or calculus—there are two conditions to be especially considered, viz., obstruction to the flow of urine and putrefactive changes. There is a tendency to accumulation and imperfect evacuation, either from impediment in front or deficient expulsive force from behind, usually from both. The putrefaction and its accompanying conditions are always present. How may these two morbid factors be attacked and overcome?

Drugs taken by the mouth can do little good in these cases; but tonics, opium, and henbane, &c., may make the disease more bearable for a time. Salol has been recommended on the grounds that it splits up in the body into its phenyl and salicyl elements, and the former pass out through the urinary tract, sometimes making the urine black. It does not, however, destroy the germs in the bladder, and, in large doses, it may further inflame the mucous membrane and do harm. Local treatment is of more avail. It should be borne in mind that the two chief requirements are: (1) Some fluid which will destroy the microbes which exist in the bladder, and (2) some means by which free drainage may be effected. Washing out the bladder with an antiseptic fluid too often fails, for reasons which will presently appear; but if energetically carried out it sometimes has a very good effect. The operation is usually performed as follows: A double canula is passed, the larger the better, and the urine drawn off. A ball syringe with a nozzle that fits tightly into the orifice of the canula is then filled with the antiseptic lotion and its contents squeezed into the bladder through the tube. Then the fluid is allowed to escape, and this must be repeated until the washings come away free from pus or mucous shreds, &c.

Lavaux recommends the use of a reservoir of fluid which is hung up about one and a half metres above the patient. When the bladder is distended (as evidenced by desire to micturate, &c.) the contents of the viscus are allowed to escape through the catheter which is used. He uses a saturated solution of boric acid, and in obstinate cases a solution of nitrate of silver. If there is much pain a 4 per cent. solution of cocaine is first introduced and allowed to remain for a few minutes.

The great disadvantage of the double canula is that the size of the canal for the escape of fluid is necessarily smaller than in an ordinary catheter, and there is more likelihood of blocking. Its passage is, moreover, often more painful than that of the soft instrument.

The drawback to the reservoir is the difficulty of regulating the pressure and knowing when the bladder is full. Probably the best plan is to use a large soft catheter and the ball syringe already mentioned. The pressure can be better regulated in this way, and the outflow is free.

But it must be remembered that the object of this operation is not only to wash out obvious deposits, such as pus and mucus, but the far more difficult one of killing and getting rid of germs; and the frequent failure which attends this treatment is not surprising when the conditions of the case are realised. For the bladder is not a round, contractile bag with smooth walls, but a cavity with recesses and irregularities from which the enemy (the microbes) can only be dislodged by an energetic and well-directed fire. The mucous membrane is rough, thickened, and sacculated by the hypertrophy of muscular bands. A glance at the outline of a corrugated bladder with enlarged prostate

FIG. 1.—Outline of walls of hypertrophied bladder. *p p*, Enlarged prostate; *d*, thickened and corrugated walls; *a*, recess behind prostate; *b*, depression between muscular bands.

(Fig. 1) will make this evident. It will be seen that behind the projecting gland (at *a*) there is a recess where pus and mucus could lodge, and where germs could breed with little fear of being disturbed. Again, in the depressions in the mucous membrane (at *b*, &c.) the same thing would happen. How may this difficulty be met? First, as to the possibility of obtaining free access to these folds and crevices. This is more effectually done if the end of the catheter is bent either in the form of a coudé or bi-coudé (Fig. 2). The sharp bend may be turned round in various directions directly it enters the bladder, and the antiseptic fluid will play against the dangerous parts at the neck of the organ where the disease lurks longest. This is done better still if the opening at the end is bevelled in such a way as to guide the outflow backwards, as in the diagram (Fig. 2 *b*).

In the next place it may be noted that the strength of the antiseptic used is rarely sufficient to act powerfully as a germicide. In a paper in *Annales des Maladies des Org. Genito-Urinaires* (10me. Année, No. 1), Professor Guyon recommends the following plan: Make a solution of perchloride of mercury with boiled distilled water of the strength of 1 in

FIG. 2.—*a*, End of coudé catheter; *b*, end of bi-coudé showing direction taken by fluid.

5,000. Do not use this as a lavage, but inject small quantities of it after the bladder has been emptied. Increase the strength until you can introduce a teaspoonful of a 1 in 1,000 solution. Do not inject more than this at a time and begin with a smaller quantity (thirty drops or so). The empty bladder can stand a stronger drug than the full one, and the germicide is much more potent when unmixed with decomposing urine. Excellent results have followed this method in France, even in cases of tuberculous cystitis.

Probably the best plan is to cleanse the organ of its ammoniacal and purulent contents by lavage with distilled water at a temperature of 100 deg. Fahr., and then follow Guyon's mode of procedure, taking care to inject a little of the antiseptic into the membranous and prostatic portions of the urethra, which parts are also affected with the disease.

Unfortunately cases often resist all treatment of this kind, and something further must be done to relieve the great misery these patients suffer.

To increase the efficiency of the drainage, the bladder has been opened above the pubes and a tube inserted. This has been followed by good results, but the disease often returns after a time, no doubt because the hypertrophied prostate, and the imperfect evacuation are not cured. Supra-pubic prostatectomy has therefore been employed. This operation (McGill's) has met with success.

In 1893 Dr. White, of Philadelphia, suggested the desirability of double castration for obstinate cases of enlarged prostate; arguing that this body and the uterus were so alike histologically that if the removal of the ovaries caused atrophy of the one, removal of the testes might cause atrophy of the other. Ramm, of Christiania, was, apparently, the first to carry out this idea, and several successful cases (Mansell Moullin, J. Swain, &c.) have since occurred. The rapidity with which the prostate withers and the cystitis improves is truly remarkable.

Other operations have been employed for special forms of cystitis, but these cannot be considered here.

## Figures and Tables

**Fig. 1. f1:**
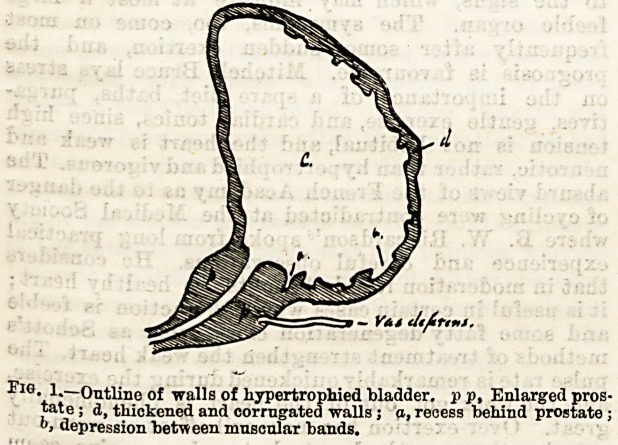


**Fig. 2. f2:**